# 改进的固相萃取-高效液相色谱法测定土壤中15种醛酮类化合物

**DOI:** 10.3724/SP.J.1123.2022.05021

**Published:** 2023-03-08

**Authors:** Kunpeng XUE, Lingyu YU, Xingfa REN, Bingfang TU, Chao CHEN, Ting XU, Huan HE, Shuai HU

**Affiliations:** 1.南京师范大学环境学院, 江苏 南京 210097; 1. School of Environment, Nanjing Normal University, Nanjing 210097, China; 2.浙江月旭材料科技有限公司, 浙江 金华 321016; 2. Zhejiang Welch Materials Co., Ltd., Jinhua 321016, China; 3.永康市质量技术监督检测中心, 浙江 金华 321300; 3. Yongkang Supervision and Inspection Center for Product Quality and Technology, Jinhua 321300, China

**Keywords:** 固相萃取, 高效液相色谱, 醛酮类化合物, 土壤, solid phase extraction (SPE), high performance liquid chromatography (HPLC), carbonyl compounds, soil

## Abstract

建立了改进的固相萃取-高效液相色谱法测定土壤中甲醛、乙醛、丙烯醛、丙酮、丙醛、丁烯醛、丁醛、苯甲醛、异戊醛、正戊醛、邻-甲基苯甲醛、间-甲基苯甲醛、对-甲基苯甲醛、正己醛和2,5-二甲基苯甲醛等15种醛酮类化合物的分析方法。利用乙腈对土壤进行超声提取,样品提取液与2,4-二硝基苯肼(2,4-DNPH)进行衍生化反应,生成稳定的腙类化合物;随后利用装有亲水亲脂平衡的*N*-乙烯基吡咯烷酮/二乙烯基苯共聚物填料的固相萃取小柱(Welchrom^®^ BRP)对衍生后的溶液进行净化;采用Ultimate^®^ XB-C18色谱柱(250 mm×4.6 mm, 5 μm)进行分离,以乙腈-水(65∶35, v/v)为流动相进行等度洗脱,于波长360 nm处进行检测,利用外标法对土壤中15种醛酮类化合物进行定量。该方法改进了环境标准HJ 997-2018《土壤和沉积物 醛、酮类化合物的测定 高效液相色谱法》中试样的处理方法,优化后得到土壤的最佳提取条件,即:提取溶剂为乙腈,提取温度为30 ℃,提取时间为10 min。结果表明:采用BRP小柱的净化效果明显优于普通硅胶基质C18小柱,15种醛酮类化合物在各自的范围内线性关系良好,线性相关系数均在0.996以上;平均加标回收率为84.6%~115.9%,相对标准偏差(RSD)为0.2%~5.1%;检出限为0.02~0.06 mg/L。该方法简便,灵敏度高,准确性好,适用于HJ 997-2018中规定的土壤和沉积物中15种醛酮类化合物的准确定量分析,为研究土壤中醛酮类化合物的残留状况和环境行为提供了可靠的技术支持。

## Abstract (en)

An improved solid phase extraction (SPE)-high performance liquid chromatography method was established to determine 15 carbonyl compounds, namely, formaldehyde (FOR), acetaldehyde (ACETA), acrolein (ACR), acetone (ACETO), propionaldehyde (PRO), crotonaldehyde (CRO), butyraldehyde (BUT), benzaldehyde (BEN), isovaleraldehyde (ISO), *n*-valeraldehyde (VAL), *o*-methylbenzaldehyde (*o*-TOL), *m*-methylbenzaldehyde (*m*-TOL), *p*-methylbenzaldehyde (*p*-TOL), *n*-hexanal (HEX), and 2,5-dimethylbenzaldehyde (DIM), in soil. The soil was ultrasonically extracted with acetonitrile, and the extracted samples were derivatized with 2,4-dinitrophenylhydrazine (2,4-DNPH) to generate stable hydrazone compounds. The derivatized solutions were cleaned using an SPE cartridge (Welchrom^®^ BRP) packed with *N*-vinylpyrrolidone/divinylbenzene copolymer. Separation was performed on an Ultimate^®^ XB-C18 column (250 mm×4.6 mm, 5 μm), isocratic elution was performed with acetonitrile-water (65∶35, v/v) as the mobile phase, and detection was performed at a wavelength of 360 nm. The 15 carbonyl compounds in the soil were then quantified using an external standard method. The proposed method improves the sample processing method described in the environmental standard HJ 997-2018: Soil and sediment-Determination of carbonyl compounds-High performance liquid chromatography. A series of experiments revealed the following optimal conditions for soil extraction: acetonitrile as the extraction solvent, extraction temperature of 30 ℃, and extraction time of 10 min. The results showed that the purification effect of the BRP cartridge was significantly better than that of the conventional silica-based C18 cartridge. The 15 carbonyl compounds showed good linearities, and all correlation coefficients were above 0.996. The recoveries ranged from 84.6% to 115.9%, the relative standard deviations (RSDs) ranged from 0.2% to 5.1%, and the detection limits were 0.02-0.06 mg/L. The method is simple, sensitive, and suitable for the accurate quantitative analysis of the 15 carbonyl compounds in soil specified in HJ 997-2018. Thus, the improved method provides reliable technical support for studying the residual status and environmental behavior of carbonyl compounds in soil.

醛酮类化合物是土壤中常见的含氧有机污染物,是重要的污染物之一。土壤中的醛酮类化合物大多具有刺激性和毒性^[1]^,该类化合物对人的眼睛、鼻子、皮肤、肺和呼吸道等均有强烈的刺激性,且有致癌、致畸等风险,危害性不容小觑^[2]^。土壤中的醛酮类化合物主要是人类活动所造成的,化工、制药、印染和农药生产等企业大量使用醛酮类化合物,尤其是甲醛被大量用作拔色剂、还原剂、消毒剂和防腐剂等的化学原料试剂,其运输、储存过程中发生的泄露以及排放的废水进入土壤和沉积物,是醛酮类化合物重要的污染来源。目前国内外制定并颁布的环境法规中均将醛酮类化合物列入重点控制的有毒有害污染物名单。2022年国务院发布的第三次全国土壤普查通知文件也将土壤中醛酮类化合物作为重点监测对象之一。

常用于检测醛酮类化合物的方法主要有高效液相色谱法(HPLC)^[3⇓⇓⇓⇓⇓⇓⇓⇓⇓-13]^、高效液相色谱-质谱法(HPLC-MS)^[14⇓⇓-17]^、气相色谱法(GC)^[18⇓-20]^和气相色谱-质谱法(GC-MS)^[21,22]^等。在这些方法中,色谱法主要是利用2,4-二硝基苯肼(2,4-dinitrophenylhydrazine, 2,4-DNPH)与醛酮类化合物结构上的羰基反应生成稳定的腙类化合物,然后经过常规的液液萃取方式进行浓缩,最后进行分离检测。该方法存在的问题是液液萃取需要消耗大量的有机溶剂,会造成二次污染,且检测灵敏度不高;采用GC-MS和HPLC-MS进行检测虽然能够极大程度地提高醛酮类化合物的检测灵敏度,但是仪器设备昂贵,实验室配置率低,质谱仪器对检测人员的技术水平要求也较高,从而导致该方法在基层实验室可操作性不强;且由于土壤基质复杂,测定时存在严重的基质干扰效应,因此检测难度也较大。环境标准HJ 997-2018^[23]^采用的检测方法主要是利用醋酸-醋酸钠溶液对土壤进行振荡提取,提取液中的醛酮类化合物在一定温度和pH下与2,4-DNPH发生衍生化反应,生成稳定的腙类化合物,利用C18小柱对样品进行净化,然后用高效液相色谱-紫外检测法对醛酮类化合物进行测定。使用该小柱净化步骤简单,并不需要经过复杂的液液萃取步骤,效果明显;该标准针对15种醛酮类化合物的方法检出限为0.02~0.06 mg/kg,定量限为0.08~0.24 mg/kg。但是,该标准中采用的检测方法存在一些问题,主要表现为以下几点:(1)提取剂用量大,样品需要用高达200 mL醋酸-醋酸钠提取溶剂进行提取,提取时间长且提取不完全;(2)衍生的溶剂体系中有机相比例极低导致衍生不完全^[24]^; (3)采用C18小柱进行前处理净化时发现,由于C18小柱是纯粹的反相疏水模式的小柱,其吸附保留、分离模式单一,很容易造成目标物吸附穿透,从而导致回收率偏低。

针对上述问题,本文采用乙腈作为提取溶剂,大大缩短了提取时间;改进了净化方式,利用具有高比表面积、大孔径和均匀孔道结构的亲水亲脂平衡的*N*-乙烯基吡咯烷酮/二乙烯基苯共聚物填料小柱进行前处理净化,由于该填料表面同时具有亲水性吡咯烷酮基团和疏水性苯基基团,能够兼顾15种醛酮类化合物的保留和土壤样品中杂质的去除效果,从而克服了HJ 997-2018方法采用C18小柱无法对15种醛酮类化合物进行有效吸附、保留等问题,获得了满意的杂质去除效果和理想的加标回收率。该方法改进了HJ 997-2018标准中存在的不足,在检测效率、准确度和重复性等方面均具有较大提升,极其适用于土壤中15种醛酮类化合物的准确定量检测。

## 1 实验部分

### 1.1 仪器、试剂与材料

Ultimate3000高效液相色谱仪(ThermoFisher公司); JP-020S超声波清洗器(功率600 W、频率40 kHz,深圳市洁盟清洗设备有限公司); XW-80A涡旋混合仪振荡器(海门市其林贝尔仪器制造有限公司); 12位真空固相萃取装置与Welchrom^®^ BRP固相萃取小柱(规格为1000 mg/6 mL)购自浙江月旭材料科技有限公司。

甲醛(FOR,纯度37%)、乙醛(ACETA,纯度40%)、丙烯醛(ACR,纯度98%)、丙酮(ACETO,纯度99%)、丙醛(PRO,纯度97%)、丁烯醛(CRO,纯度99%)、丁醛(BUT,纯度99%)、苯甲醛(BEN,纯度99%)、异戊醛(ISO,纯度97%)、正戊醛(VAL,纯度97%)、邻-甲基苯甲醛(*o*-TOL纯度97%)、间-甲基苯甲醛(*m*-TOL纯度97%)、对-甲基苯甲醛(*p*-TOL纯度97%)、正己醛(HEX,纯度98%)、2,5-二甲基苯甲醛(DIM,纯度99%),购于德国Merck公司;15种醛酮类化合物-DNPH混合标准溶液(甲醛-DNPH、乙醛-DNPH、丙烯醛-DNPH、丙酮-DNPH、丙醛-DNPH、丁烯醛-DNPH、丁醛-DNPH、苯甲醛-DNPH、异戊醛-DNPH、正戊醛-DNPH、邻-甲基苯甲醛-DNPH、间-甲基苯甲醛-DNPH、对-甲基苯甲醛-DNPH、正己醛-DNPH和2,5-二甲基苯甲醛-DNPH,以醛、酮类化合物计的质量浓度均为100 mg/L),购自上海安谱实验科技股份有限公司。乙腈(色谱纯,德国Merck公司); 2,4-DNPH(色谱纯,上海麦克林生化科技有限公司);磷酸(分析纯,上海阿拉丁生化科技股份有限公司)。实验用水为娃哈哈超纯水(色谱级,杭州娃哈哈集团有限公司)。

主要试验材料为土壤,所有样品均采集于金华市婺城区白龙桥附近不同区域的土壤。

### 1.2 标准溶液的配制

系列标准溶液:将100 mg/L醛酮-DNPH混合标准溶液用乙腈配制成质量浓度为0.1、0.2、0.5、2、5、10和20 mg/L的系列标准溶液。

DNPH衍生剂:准确称取0.3 g 2,4-DNPH粉末,用乙腈溶解定容至100 mL,质量浓度为3 g/L。

### 1.3 样品的提取和衍生

提取:首先,去除土壤样品中的异物(石子、叶片等),称取10 g土壤样品于50 mL离心管中,准确加入25 mL乙腈,涡旋混匀,30 ℃条件下超声分散提取10 min, 4000 r/min离心5 min,收集上清液;然后准确加入15 mL乙腈,重复提取一次,4000 r/min离心5 min,收集上清液;第三次准确加入10 mL乙腈重复提取,4000 r/min离心5 min,收集上清液。合并3次提取后的上清液,待衍生。

衍生:准确移取25 mL样品提取液,加入6 mL的DNPH衍生剂,1 mL质量分数为20%的磷酸溶液,于4 ℃条件下衍生6 h,加入100 mL水稀释,待净化。

### 1.4 样品的净化

采用BRP小柱对样品进行净化,分为以下4个步骤:(1)活化:向BRP固相萃取小柱中依次加入10 mL乙腈和10 mL 20%(v/v)乙腈水溶液,弃去流出液;(2)上样:将待净化溶液加入活化后的小柱中,控制流速为2~3滴/s,弃去流出液;(3)淋洗:加入10 mL 20%乙腈水溶液,弃去流出液;(4)洗脱:加入9 mL乙腈洗脱小柱,收集洗脱液至10.0 mL容量瓶中,并用乙腈定容至10 mL,待检测。

### 1.5 液相色谱条件

色谱柱:月旭Ultimate^®^ XB-C18色谱柱(250 mm×4.6 mm, 5 μm);流动相:乙腈-水(65∶35, v/v);流速:1.0 mL/min;柱温:30 ℃;进样量:20 μL;检测波长:360 nm。

### 1.6 基质匹配标准溶液的配制

按照1.4节的步骤,采用SPE小柱对空白样品进行前处理,收集乙腈洗脱液作为空白基质溶液,配制质量浓度为0.1、0.2、0.5、2、5、10和20 mg/L的基质匹配标准溶液。

## 2 结果与讨论

### 2.1 色谱条件的优化

HJ 997-2018标准采用的液相色谱流动相条件为乙腈-水(60∶40, v/v),该标准给定的参考色谱图见图1。可以看出共有3组峰无法基线分离,分别是ACR和ACETO、*m*-TOL和*p*-TOL、HEX和DIM。本文考察了流动相中乙腈的比例对15种醛酮类化合物分离的影响。将乙腈的比例分别调整为60%、65%和68%,考察15种醛酮类化合物的分离效果。结果表明15种醛酮类化合物的分离度对流动相中有机相的比例极为敏感,如图2所示。15种醛酮类化合物在3种不同乙腈比例下,仍然有两组目标物无法基线分离,分别是ACR和ACETO、*m*-TOL和*p*-TOL,但是,HEX和DIM却完全能够基线分离,分离度均超过1.5,明显优于HJ 997-2018标准给定色谱图的分离效果。此外,流动相乙腈的比例分别为65%和68%时,ACR和ACETO、*m*-TOL和*p*-TOL这两组目标物的分离度类似,65%乙腈对15种醛酮类化合物的整体分离效果更好,且15种醛酮类化合物的保留时间均在30 min以内,因此本文选择65%乙腈水溶液作为最优的流动相。

**图1:**
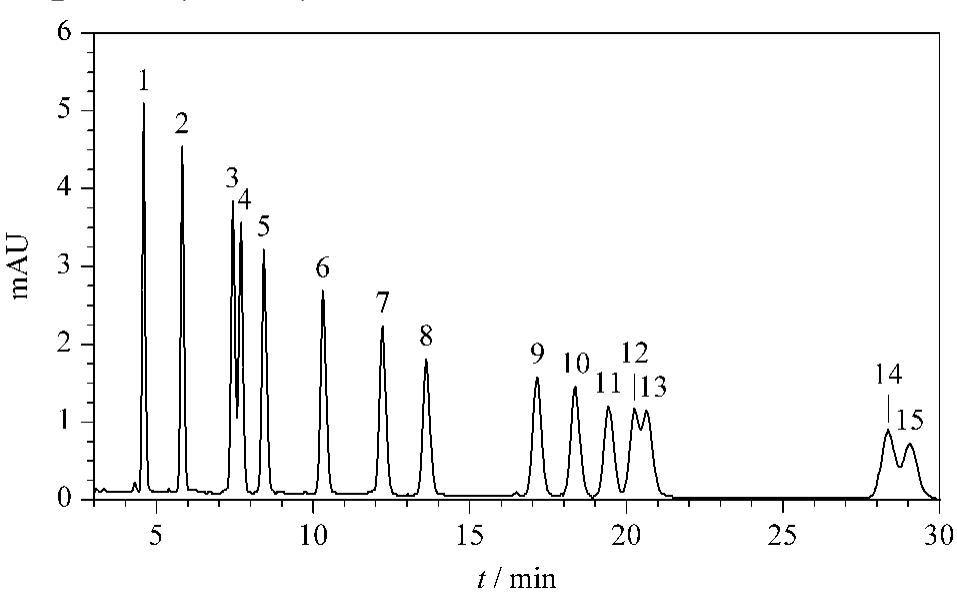
HJ 997-2018标准中15种醛酮-DNPH衍生物的色谱图^[23]^

**图2:**
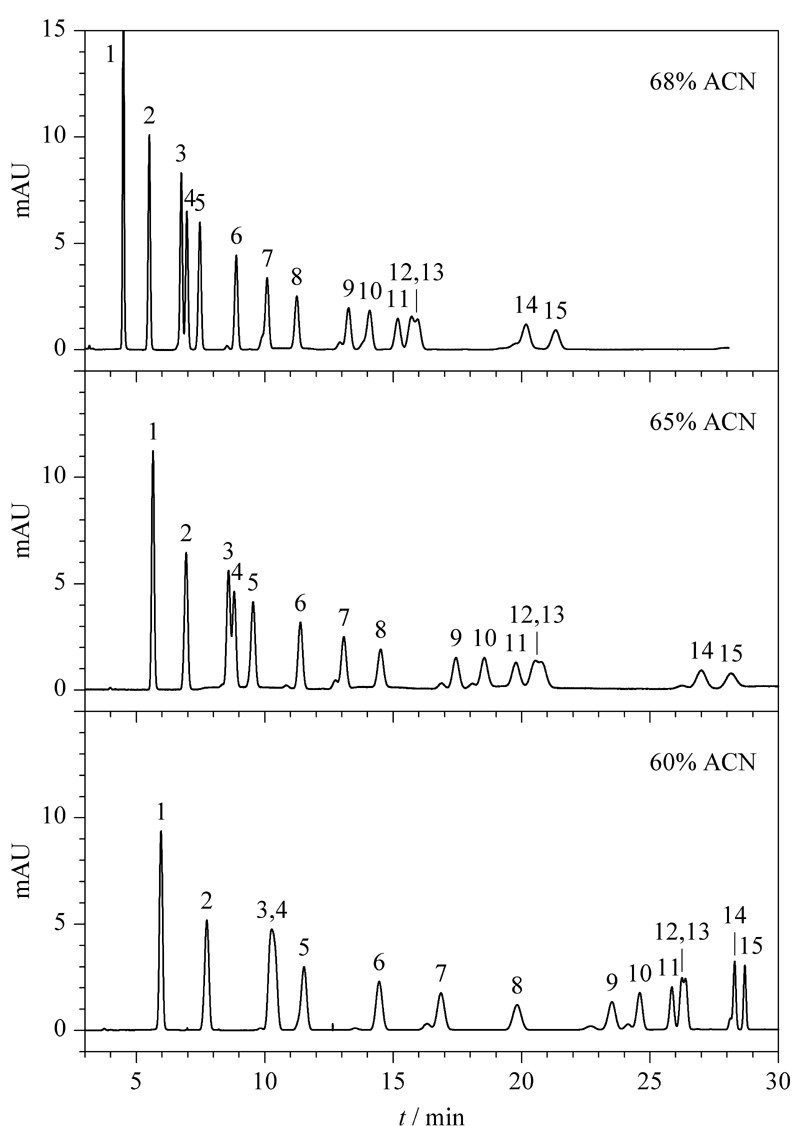
15种醛酮-DNPH衍生物标准溶液的色谱图

### 2.2 衍生试剂的选择

HJ 997-2018标准采用的衍生方法具体为:准确移入采用醋酸-醋酸钠缓冲液提取的样品提取液100 mL于平底烧瓶中,加入4 mL pH 3.0柠檬酸-柠檬酸钠缓冲溶液、6 mL 2,4-DNPH乙腈溶液,置于恒温振荡器中,40 ℃振荡1 h。该衍生体系中水和乙腈的比例约为17∶1,水分的含量极高,而2,4-DNPH试剂微溶于水,从而造成衍生后有明显的絮状沉淀析出,且衍生产物腙类化合物在高比例的水中不稳定^[24]^,如图3a所示。增加衍生体系中乙腈的比例,当水和乙腈比例为10∶1时,仍然可以看到有部分絮状沉淀出现,如图3b所示。因此,本文改进了HJ 997-2018标准中的衍生方式,在衍生过程中增加乙腈的比例,具体为:准确移取100 mL用乙腈提取的样品提取液,依次加入6 mL 2,4-DNPH乙腈溶液衍生试剂和1 mL 20%磷酸溶液后于4 ℃冷藏衍生6 h,结果表明没有絮状沉淀析出,如图3c所示。该结果也进一步证明了乙腈作为土壤的提取溶剂是合适的,不仅有利于土壤基质中目标物提取完全,同时也确保了醛酮类化合物在高比例有机相衍生溶剂体系下与2,4-DNPH衍生完全,不产生絮状沉淀。

**图3:**
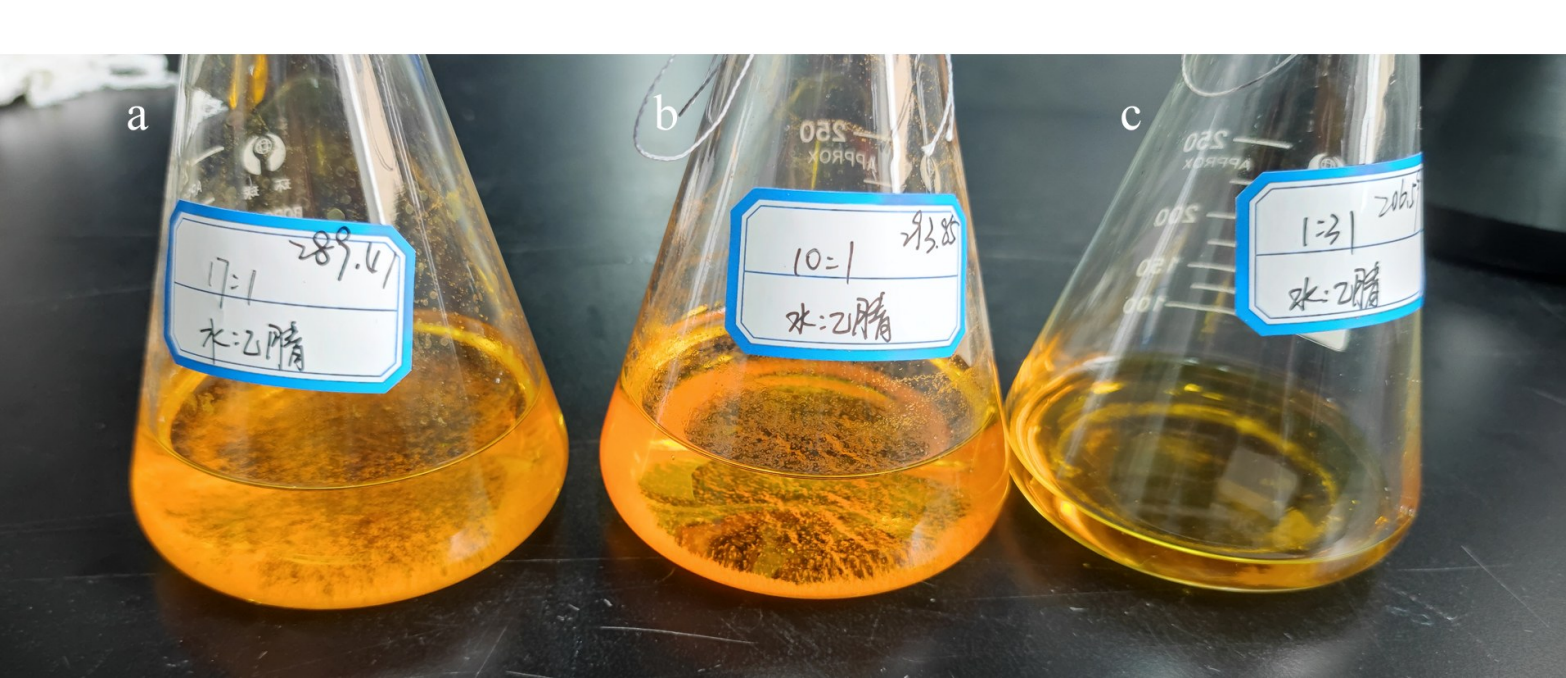
不同溶剂体系对15种醛酮类化合物的衍生效果

### 2.3 提取与净化条件考察

#### 2.3.1 提取溶剂的选择

按照HJ 997-2018标准推荐的方法进行土壤中15种醛酮类化合物提取效率的考察。具体步骤为:采用醋酸-醋酸钠溶液作为提取液,经2,4-DNPH衍生、C18小柱净化后测定其提取回收率,结果如图4所示。采用该提取方法时*o*-TOL、*m*-TOL、*p*-TOL和HEX的提取效率为0,且除FOR以外的其他醛酮类化合物的提取效率均在65.6%以下。针对这种情况,本文改变提取溶剂的种类进行提取,分别选用50 mL 10%乙腈水溶液、50 mL 50%乙腈水溶液以及50 mL乙腈分别提取3次,结果表明:采用10%乙腈水溶液和50%乙腈水溶液进行提取均有多种醛酮类化合物无法提取完全,*m*-TOL和*p*-TOL的提取效率仍然为0,且DIM的提取效率也仅为28.6%,处于较低水平。而采用乙腈的提取效果不但溶剂用量少,仅为标准方法提取溶剂体积的1/4,且提取效率高,加标回收率为64.5%~135.3%。原因可能是利用有机溶剂作为提取溶剂,依据相似相溶原理,乙腈能够高效地将醛酮类化合物从土壤中萃取出来。

**图4:**
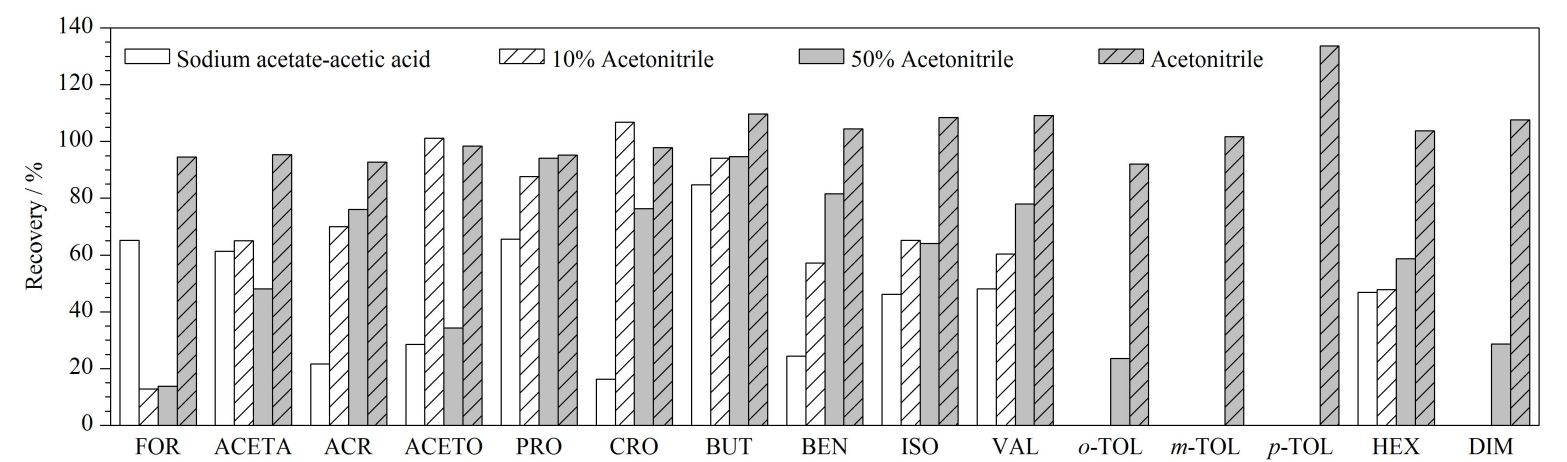
提取溶剂对15种醛酮类化合物提取效率的影响

#### 2.3.2 提取温度的选择

实验过程中考察了20、30、40、50、60和70 ℃等不同提取温度对醛酮类化合物提取效率的影响,结果见图5。提取温度低于30 ℃时,15种醛酮类化合物的提取效率较为稳定,均在80.0%以上。提取温度超过30 ℃时,大部分醛酮类化合物的提取效率降低,原因可能是15种醛酮类化合物大多是挥发性有机污染物,当提取温度较高时会因为挥发造成损失,从而导致提取效率偏低。综合考虑,本文选用提取温度为30 ℃。

**图5:**
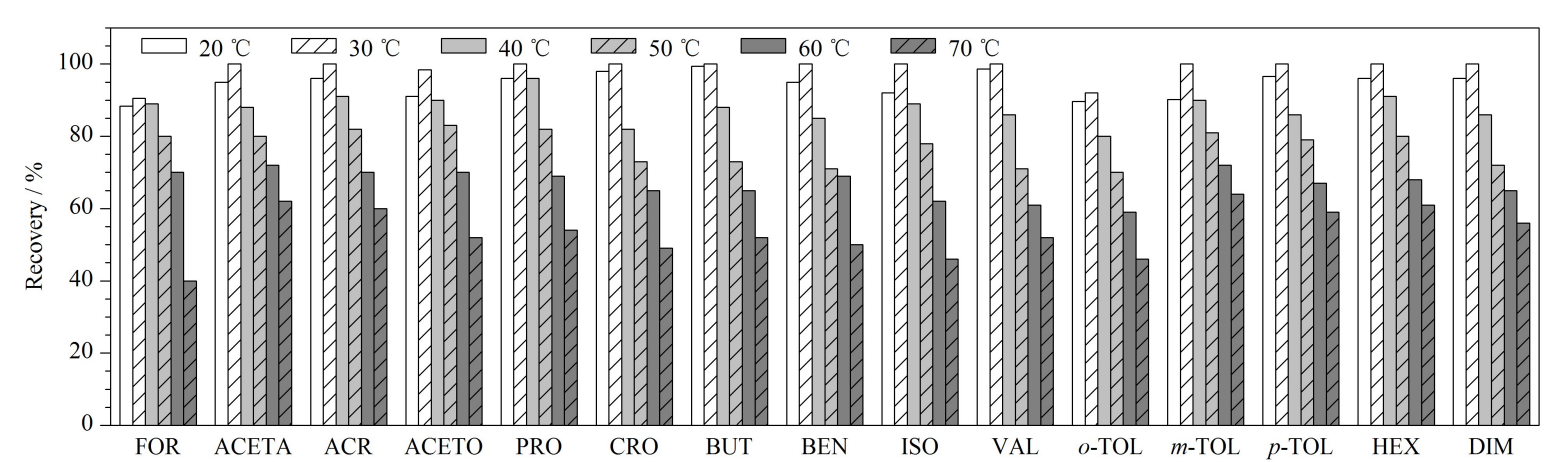
提取温度对15种醛酮类化合物提取效率的影响

#### 2.3.3 样品提取时间的选择

HJ 997-2018标准给定的样品提取时间为不少于18 h,提取时间较长。因此,本文考察了5、10、20、40和60 min等不同提取时间对醛酮类化合物提取效率的影响,结果见图6。

**图6:**
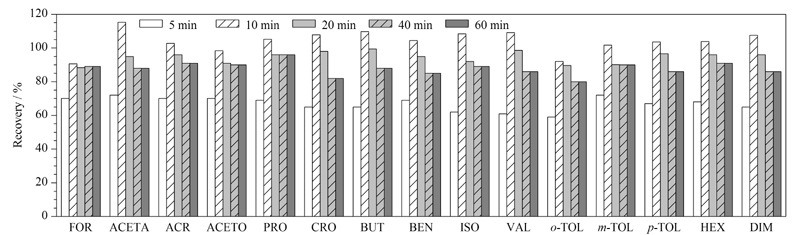
提取时间对15种醛酮类化合物提取效率的影响

由图6可以看出,提取10 min时,提取效率达到最高,15种醛酮类化合物基本在80%以上,之后趋于稳定。原因可能是乙腈作为有机溶剂萃取目标物的能力强,可以将醛酮类化合物快速地从土壤中萃取出来,从而缩短提取时间。

#### 2.3.4 样品净化小柱的选择及优化

按照HJ 997-2018的要求,用商品化的反相SPE柱对多种土壤进行前处理净化。分别考察了使用Welchrom^®^ C18小柱、Welchrom^®^ PS-DVB小柱和Welchrom^®^ BRP小柱(规格均为1000 mg/6 mL)等3种不同类型的SPE小柱的净化效果。结果表明:采用BRP小柱进行净化后15种醛酮类化合物的加标回收率均在80.1%以上,而C18小柱和PS-DVB小柱有相当一部分目标物的加标回收率在70.0%以下。其中,C18小柱对ISO和VAL的加标回收率分别为62.4%和70.3%; PS-DVB小柱对ISO和VAL的加标回收率分别为71.7%和72.1%。原因可能是BRP和PS-DVB聚合物基质小柱比表面积远高于硅胶基质C18小柱,粒径相比硅胶基质填料来说也更大,因而吸附容量更大,目标物在填料中扩散得更快,不会造成小柱堵塞;并且,BRP和PS-DVB填料的平均孔径更大、孔道结构分布比较均一,达到20 nm,而C18填料平均孔径仅为4 nm,因而聚合物基质填料小柱能够促使目标物较好地保留且与土壤基质中的杂质较好地分离,3种小柱的填料理化参数如表1所示,SEM图谱如图7所示,可以明显看出PS-DVB和BRP填料是相对均一的球型形貌,而C18填料存在一些不规则的颗粒,因此由于传质阻力的增大,导致其保留目标物和去除杂质的效果不如球形的聚合物填料。而BRP小柱的加标回收率优于PS-DVB小柱的原因可能是: BRP小柱填料是兼顾亲水亲脂平衡的分离材料,它可以通过反相疏水、空间位阻、氢键和离子交换等多种化学作用力对15种醛酮类化合物均具有较强的化学作用力,从而使得15种醛酮类化合物具有较高的加标回收率;而PS-DVB只能通过纯粹的疏水作用力和目标物产生相互作用,无法兼顾结构相似的醛酮目标物(例如*o*-TOL、*m*-TOL和*p*-TOL等)的保留和分离。

**表1:** 3种反相固相萃取小柱的填料理化参数

SPE column type	Stationary phase group	Morphology	Specific surface area/(m^2^/g)	Particle size/μm	Pore size/nm
C18	octadecyl	amorphous	500	40-63	4
PS-DVB	polystyrene-divinyl polymer	spherical	700	50-70	20
BRP	N-vinylpyrrolidone-divinyl polymer	spherical	700	50-70	20

**图7:**
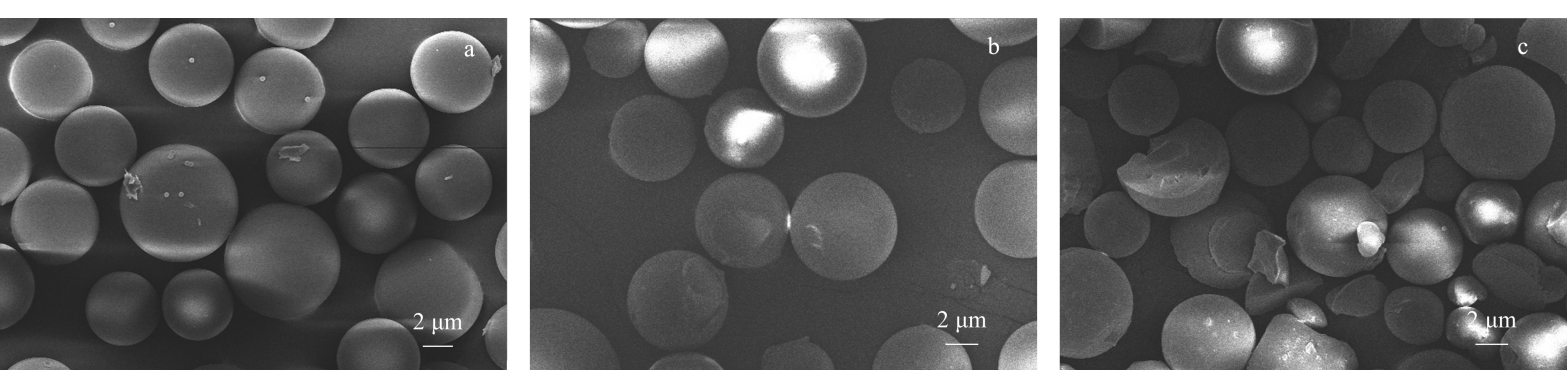
3种固相萃取小柱填料的SEM形貌图

对1.6节配制的基质匹配溶液和与其具有相同浓度的标准溶液在同等条件下进行测定,比较二者的峰面积,用于评价3种固相萃取小柱净化方式的基质效应(ME),具体见公式(1)^[25]^。


(1) $ME=\frac{A_{m}-A_{b}}{A_{s}}\times 100\%$

式中,*A*_m_、*A*_b_和*A*_s_分别为基质匹配标准溶液、空白基质溶液和标准溶液中目标物的峰面积。3种不同类型的小柱净化对15种醛酮目标物基质效应结果见表2所示。可以看出:采用C18小柱对15种醛酮类化合物的ME值为110.4%~145.6%, PS-DVB小柱对15种醛酮类化合物的ME值为108.7%~132.1%, BRP小柱对15种醛酮类化合物的ME值为90.5%~109.7%,进一步说明了BRP小柱能够最大限度地降低基质效应,提高分析测试结果的准确度,满足其定量要求。

**表2:** 3种SPE填料对15种醛酮类化合物基质效应的影响(*n*=6)

Compound	C18			PS-DVB			BRP	
	ME/%	RSD/%		ME/%	RSD/%		ME/%	RSD/%
FOR	110.4	1.8		108.7	1.2		90.5	1.3
ACETA	125.6	3.4		129.0	3.6		115.9	3.1
ACR	113.9	2.8		110.2	2.1		102.7	4.6
ACETO	129.0	2.7		118.0	3.5		98.4	1.3
PRO	112.8	2.4		116.1	2.4		105.2	2.3
CRO	127.2	4.6		125.4	3.6		107.8	1.0
BUT	145.4	5.6		132.1	5.3		109.7	2.2
BEN	128.0	3.7		127.1	2.0		104.4	1.3
ISO	120.9	4.1		123.7	4.7		108.3.	2.8
VAL	130.5	4.3		124.3	4.4		109.1	3.0
o-TOL	115.7	3.2		118.6	3.5		92.0	1.7
m-TOL	113.3	4.0		113.7	3.0		101.7	2.5
p-TOL	117.3	4.2		118.5	3.7		103.6	1.3
HEX	111.2	1.6		116.4	1.9		103.8	1.3
DIM	115.6	1.8		118.9	2.3		107.6	1.6

### 2.4 方法学考察

#### 2.4.1 线性范围与检出限

准确配制0.1、0.2、0.5、1.0、2.0、5.0、10和20 mg/L的标准系列溶液,分别进样20 μL,采用峰面积定量,测定结果进行线性回归,线性曲线方程(*y*为峰面积,*x*为质量浓度,mg/L)见表3,相关系数为0.996以上,可以满足定量的要求。以信噪比为3和10时样品的质量浓度作为方法的检出限(LOD)和定量限(LOQ), 15种醛酮类化合物的LOD为0.02~0.06 mg/L,LOQ为0.10~0.20 mg/L。15种醛酮类化合物的LOD和LOQ大多优于HJ 997-2018中规定的结果。

**表3:** 15种醛酮类化合物的线性关系、检出限和定量限

Compound	Linear range/(μg/mL)	Linear equation	r^2^	LODs/(mg/L)			LOQs/(mg/L)	
				This method	HJ 997-2018		This method	HJ 997-2018
FOR	0.1-20.0	y=13.613x+0.052	0.997	0.02	0.02		0.05	0.08
ACETA	0.2-20.0	y=10.242x+0.045	0.996	0.03	0.04		0.10	0.16
ACR	0.2-20.0	y=9.355x+0.038	0.996	0.03	0.04		0.10	0.16
ACETO	0.2-20.0	y=7.977x+0.040	0.997	0.03	0.04		0.10	0.16
PRO	0.2-20.0	y=7.419x+0.017	0.996	0.03	0.04		0.10	0.16
CRO	0.2-20.0	y=6.648x+0.017	0.997	0.04	0.04		0.14	0.16
BUT	0.5-20.0	y=4.789x+0.005	0.998	0.04	0.04		0.13	0.16
BEN	0.2-20.0	y=4.633x+0.024	0.996	0.04	0.06		0.13	0.24
ISO	0.5-20.0	y=4.037x+0.015	0.996	0.05	0.06		0.18	0.24
VAL	0.5-20.0	y=3.558x+0.067	0.997	0.05	0.06		0.18	0.24
o-TOL	0.5-20.0	y=3.865x+0.012	0.996	0.05	0.05		0.10	0.20
m-TOL	0.5-20.0	y=4.113x+0.014	0.999	0.05	0.06		0.18	0.24
p-TOL	0.5-20.0	y=3.588x+0.025	0.999	0.05	0.06		0.18	0.24
HEX	0.5-20.0	y=3.931x-0.047	0.998	0.06	0.06		0.20	0.24
DIM	0.5-20.0	y=3.294x-0.003	0.999	0.06	0.06		0.20	0.24

*y*: peak area; *x*: mass concentration, mg/L.

#### 2.4.2 准确度和精密度

在空白土壤样品中加入一定浓度的醛酮类化合物标准溶液,使得样品中醛酮类化合物的含量分别为0.2、0.5和2.0mg/kg,每个添加水平做6个平行试验,按照上述优化的实验方法进行加标回收率试验,计算平均回收率和相对标准偏差(RSD),如表4所示。15种醛酮类化合物的平均加标回收率为84.6%~115.9%,RSD为0.2%~5.1%。标准方法的加标回收率测定结果普遍在80%以下,充分说明本方法的准确度和精密度良好。

**表4:** 15种醛酮类化合物的加标回收率及RSD (*n*=6)

Compound	0.2 mg/kg			0.5 mg/kg			2.0 mg/kg		
	Recovery/%	RSD/%		Recovery/%	RSD/%		Recovery/%	RSD/%	
FOR	84.6	1.7		88.3	2.6		90.5	1.3	
ACETA	112.7	4.1		110.2	4.1		115.9	4.1	
ACR	99.3	5.0		106.9	2.8		102.7	4.6	
ACETO	104.4	1.2		101.1	1.7		98.4	1.3	
PRO	105.1	3.5		106.1	2.7		105.2	2.3	
CRO	107.2	1.6		108.4	2.5		107.8	1.0	
BUT	100.2	3.5		99.8	3.6		109.7	0.2	
BEN	106.3	3.9		105.4	2.7		104.4	1.3	
ISO	107.0	4.3		110.6	3.0		108.3	2.8	
VAL	105.2	1.9		98.6	1.6		109.1	3.0	
o-TOL	87.5	0.6		89.7	3.6		92.0	1.7	
m-TOL	85.4	2.8		90.2	2.7		101.7	2.5	
p-TOL	91.2	5.1		96.6	2.9		103.6	1.3	
HEX	104.0	2.9		106.5	2.5		103.8	0.3	
DIM	97.6	2.0		96.1	1.8		107.6	0.6	

### 2.5 实际土壤样品的测定

选取10份金华市婺城区城乡接合部工业园区土壤样品, 按照优化的实验方法进行测定。结果表明:本次采集的土壤中甲醛、乙醛、丙酮和苯甲醛这4种醛酮类化合物均有检出,其中甲醛的最大残留量为0.94 mg/kg,乙醛的最大残留量为1.53 mg/kg,丙酮的最大残留量为1.34 mg/kg,苯甲醛的最大残留量为1.36 mg/kg,充分表明土壤中存在的这4种醛酮类化合物值得重点关注。

## 3 结论

本文建立了改进的固相萃取-高效液相色谱法快速测定土壤中15种醛酮类化合物的方法。方法线性范围、检出限、精密度、准确度以及实际样品测定均取得了满意结果,表明本方法具有简单准确、检测效率高等特点。与现有的环境标准HJ 997-2018标准方法相比,该方法具有样品提取效率高、节省试剂、灵敏度和准确度高、回收率高、重复性好等优点,可以满足大批量土壤样品中15种醛酮类化合物的快速、同时检测。
